# Changes of the Occlusal Relationship in Patients With Temporomandibular Disorders (TMD) After Manual Therapy: A Pilot Study

**DOI:** 10.1002/cre2.70147

**Published:** 2025-05-19

**Authors:** Tobias Dieter Peter Pohl, Alessia Celine Harhoff, Johannes Ries, Manfred Wichmann, Ragai‐Edward Matta

**Affiliations:** ^1^ Department of Prosthodontics (Head: Prof. Dr. Manfred Wichmann) Erlangen University Hospital Erlangen Germany

**Keywords:** diagnosis, manual therapy, occlusal changes, occlusion, temporomandibular disorders, treatment

## Abstract

**Objectives:**

Temporomandibular disorders (TMD) are often linked to changes in dental occlusion, yet the impact of therapeutic interventions remains unclear. This pilot study investigates the effects of manual therapy on occlusion in TMD patients through quantitative analysis of intraoral scans.

**Material and Methods:**

Ten individuals were diagnosed with TMD and underwent a 40‐min session of manual therapy (Group MT). Ten subjects were allocated to the healthy control group (Group C) and did not receive any therapy during the 10‐week control period. Occlusion measurements were obtained using the TRIOS 3 intraoral scanner (3Shape, Denmark) both before and after the therapy or control period. The digital models were analyzed regarding occlusal changes using the GOM Inspect Professional software (GOM, Germany). The differences in the individual axes ${\mathrm{dX}}_{\mathrm{Pat}},{\mathrm{dY}}_{\mathrm{Pat}},{\mathrm{dZ}}_{\mathrm{Pat}}\;\left(\mathrm{mm}\right)$ and Euclidean distance ${dXYZ}_{\mathrm{Pat}}\;(\mathrm{mm})$ were computed.

**Results:**

In terms of the arithmetic mean, Group MT exhibited higher deviations across all three axes (${\mathrm{dX}}_{PatMT}=0.122\;(\mathrm{mm}),{\mathrm{dY}}_{PatMT}=0.217\;(\mathrm{mm}),\;{\mathrm{dZ}}_{PatMT}\;=\;0.193\;(\mathrm{mm})$) as well as in the Euclidean distance $\left({dXYZ}_{PatMT}=0.347\;(\mathrm{mm})\right)$ than the control group $\left(dX_{PatC}=0.060\;(\mathrm{mm}),\;dY_{PatC}=0.063\;(\mathrm{mm}),\;dZ_{PatC}\;=\;0.043\;(\mathrm{mm}),\;dXYZ_{PatC}=0.113\;(\mathrm{mm})\right)$. Statistically significant differences were observed for the *Y* and *Z* axes, as well as the Euclidean distance (*p* < 0.05).

**Conclusion:**

Considering the limitations of this pilot study, it is reasonable to suggest that manual therapy has a significant influence on occlusion in habitual intercuspidation among TMD patients.

## 1 Introduction

Temporomandibular dysfunction (TMD) includes all disorders affecting the temporomandibular joints, masticatory muscles, and associated tissues (Greene 2010). The clinical symptoms of these disorders manifest in various ways and can occur independently or in combination (Durham et al. 2015). In addition to joint noises (Scrivani et al. 2008) and functional limitations, individuals commonly experience acute or persistent pain (Greene 2010).

The prevalence of TMD is approximately 34% in the world population, while the peak incidence occurs between the ages of 18 and 60 (Zieliński et al. 2024). Among children the prevalence is around 11% (Valesan et al. 2021). Additionally, women are more commonly affected than men (Bueno et al. 2018; Gonçalves et al. 2010).

The treatment options for TMD are categorized based on their invasiveness and reversibility, with reversible and noninvasive therapies generally recommended as the initial treatment approach (Durham et al. 2015; Greene 2001; Liu and Steinkeler 2013).

In addition to providing information about the disorder, behavioral therapy and medication (particularly analgesics) (Durham et al. 2015), physiotherapy (including manual therapy) and occlusal splint therapy are utilized as reversible and conservative initial treatment options (Villar‐Aragón‐Berzosa et al. 2024).

The results of Gesslbauer et al.'s pilot study also support the use of osteopathic manipulative treatment and osteopathy in the cranial field for the treatment of TMD (Gesslbauer et al. 2018).

Manual therapy, a specialized field within physiotherapy, is primarily used for treating spinal symptoms (de Melo et al. 2020). The goals of manual therapy include improving range of motion, decreasing muscle spasm, and reducing pain through various mobilization, manipulation, and massage techniques (Bialosky et al. 2009). In the context of TMD therapy, this may involve mobilization of the temporomandibular joint and muscles, active or passive stretching exercises, and guided mandibular movements (de Melo et al. 2020). Due to the multifactorial etiology and complexity of the condition, cervical spine manipulation and mobilization, soft tissue techniques, and massage of the masticatory and cervical muscles may also be utilized (Villar‐Aragón‐Berzosa et al. 2024). However, the exact mechanism of action of manual therapy is not yet fully understood (Bialosky et al. 2009).

The recent review by Vieira et al. supports manual therapy as effective for TMD (Vieira et al. 2023). Manual therapy provides the best posttreatment pain reduction within observation periods of up to 5 months (Al‐Moraissi et al. 2022). In the field of symptom control and improvement in mandibular movements, upper cervical manipulation and mobilization have shown the strongest evidence (Calixtre et al. 2015). On the other hand, the existing evidence is considered very limited in a present review by Asquini et al. with only a slight superiority over other therapies in two of six analyzed studies (Asquini et al. 2022).

The etiology of TMD has been a topic of ongoing debate (Durham et al. 2015). Over the years, biomedical, psychological and biopsychosocial models have been established (Suvinen et al. 2005). Utilizing the biopsychosocial health model for the assessment of TMD patients is the most suitable approach (González‐Sánchez et al. 2023). In addition to biological factors, behavioral, environmental as well as emotional and cognitive factors need to be considered (Scrivani et al. 2008).

The role of occlusion as an etiological factor within these models has also been a subject of debate: while older publications described malocclusion as an important trigger for TMD (Upton et al. 1984), recent systematic reviews have shown that only two out of almost 40 investigated occlusal changes or abnormalities are predominantly associated with TMD (Manfredini et al. 2017). Therefore, it cannot be assumed that malocclusion triggers TMD (Luther 2007; Stone et al. 2017). Its influence on the development of the disorder is considered to be minimal to very minimal (Kalladka et al. 2022). However, individual clinical studies, such as the one by Guo et al. in 2025, show that patients with sagittal asymmetry, asymmetrical missing teeth, or asymmetrical contact weight have a higher prevalence of TMD (Guo et al. 2025).

But conversely, persistent TMD disorders can indeed have an impact on occlusion (Kalladka et al. 2022; Caldas et al. 2016). Thus, abnormal occlusal conditions are more likely to be consequences rather than causes of TMD (Türp et al. 2008).

It is also known that a combination of splints and physiotherapy affects the craniomandibular and craniovertebral relation in TMD patients (Derwich et al. 2022). Changes in occlusal conditions due to splint therapy have also been observed in patients with bruxism (Fujii et al. 2005).

The extent to which manual therapy alone can induce changes in a patient's habitual intercuspidation and the magnitude of these changes have not yet been quantitatively investigated.

## 2 Materials and Methods

The null hypothesis assumes that there are no significant changes in dental occlusion resulting from manual therapy in TMD patients as compared to the healthy control group.

This study was approved by the Ethics Committee of the University Hospital Erlangen (Reference number 233_19 B) and follows the principles of the Declaration of Helsinki (World Medical Association Declaration of Helsinki 2013).

Furthermore, the study is registered in the German Clinical Trials Register (DRKS) of the Federal Institute for Drugs and Medical Devices (BfArM) (ID DRKS00030298).

The data for this study were collected between July 2019 and January 2022 at the Department of Dentistry 2 at the University Hospital Erlangen.

### 2.1 Groups

The following exclusion criteria were applied: minority, currently undergoing orthodontic treatment, previous surgeries in the temporomandibular joint, cervical spine or adjacent structures. Patients with an existing TMD diagnosis or prior TMD therapy were also excluded.

All study participants received an information sheet about the study and signed an informed consent form and a data protection declaration before the study began. The participants did not receive any compensation for their involvement.

All subjects were examined using the TMD short examination as described by Ahlers and Jakstat (Ahlers and Jakstat 2015). This can be performed without special instruments and evaluates six criteria, each of which can be answered with a simple “yes” or “no.” These include asymmetric mouth opening, restricted mouth opening, joint sounds, occlusal sounds, painful muscle palpation, and traumatic eccentricity. If there are two or more positive findings, the presence of TMD is likely (Ahlers and Jakstat 2015).

Based on the aforementioned criteria, 10 participants were diagnosed with TMD and were assigned to the manual therapy group (Group MT). The remaining 10 participants exhibited only 1 or no signs of TMD according to the TMD short examination and were assigned to the healthy control group (Group C).

Group Manual Therapy (MT) – ø age: 40 years (23–77 years), 60% female, 40% male

**Figure 1:**
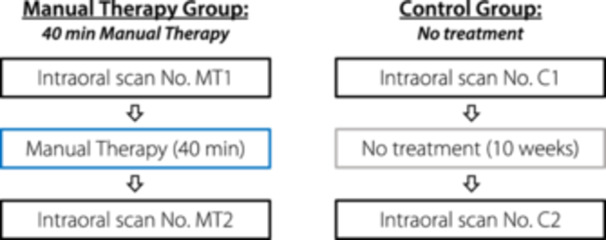
Flowchart Manual Therapy Group (MT) versus Control Group (C).

These participants received a single 40‐min session of manual therapy (MT). Intraoral scans were conducted immediately before and after the MT session (see Figure 1).

The manual therapy was consistently performed by the same physiotherapist.

Control Group/No Treatment (C) – ø age: 23 years (21–26 years), 50% female, 50% male

In this group, two intraoral scans were conducted with a 10‐week interval (see Figure 1). The healthy participants did not receive any therapy during this time.

### 2.2 Intraoral Scans and Analysis

The occlusion was captured using the digital intraoral scanner TRIOS3 (3Shape, Copenhagen, Denmark).

During the initial scan of each subject, both jaws were fully captured. The occlusion was registered in habitual intercuspidation from buccal on both sides. Both the molar and premolar regions on both sides were included in the scan. Additionally, attention was given to ensuring the patient's upright sitting position. After completing the therapy or the control period, the occlusion was newly captured buccally on both sides for each subject.

All intraoral scans were automatically post‐processed by the TRIOS 3 and then exported as Standard Tessellation Language (STL) files.

Further analysis was conducted using GOM Inspect Professional software (GOM, Braunschweig, Germany), following closely a previous study by Wolf et al (Wolf et al. 2019).

As it is common in coordinate measurement technology, a nominal model was compared to an actual model to determine three‐dimensional deviations. One model included both the upper and lower jaw scans, as well as their relation (occlusal scan) to each other.

**Figure 2:**
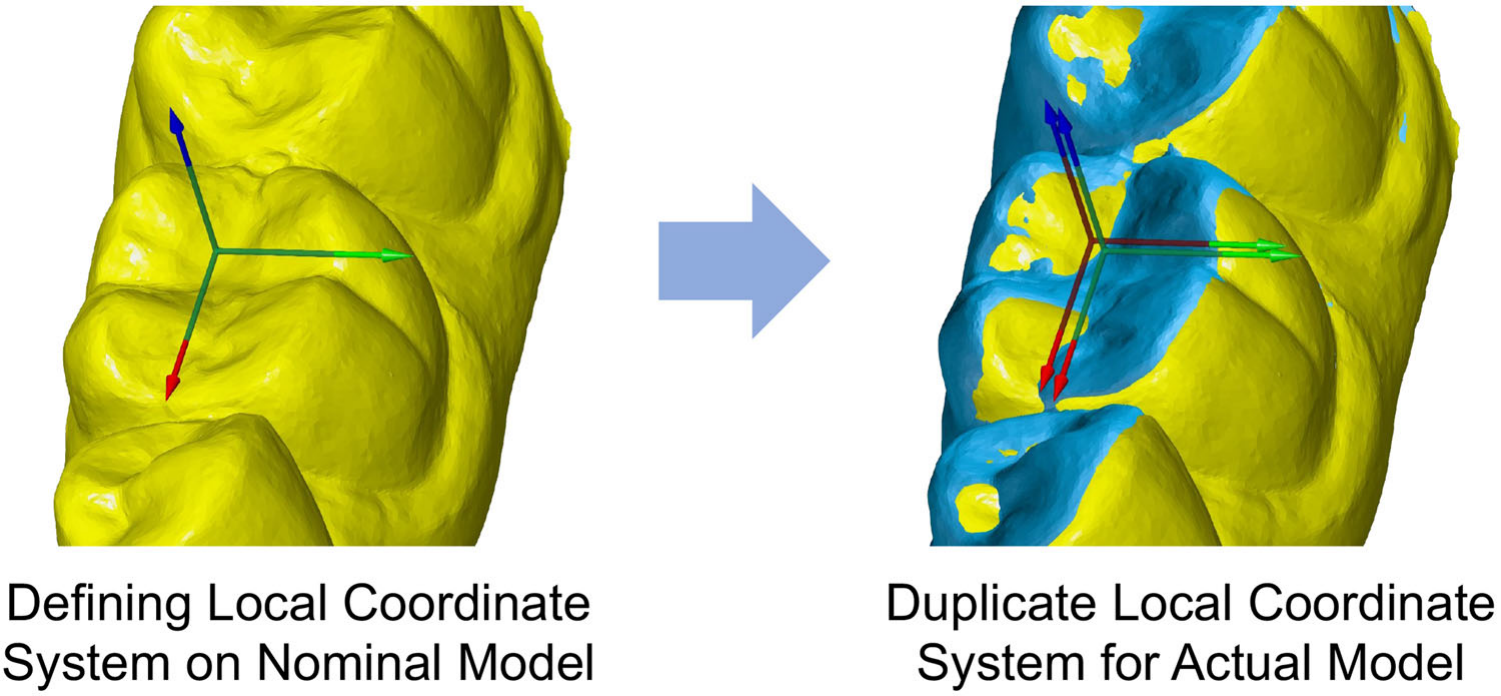
Defining local coordinate systems for the nominal (yellow) and the actual (blue) model.

**Figure 3:**
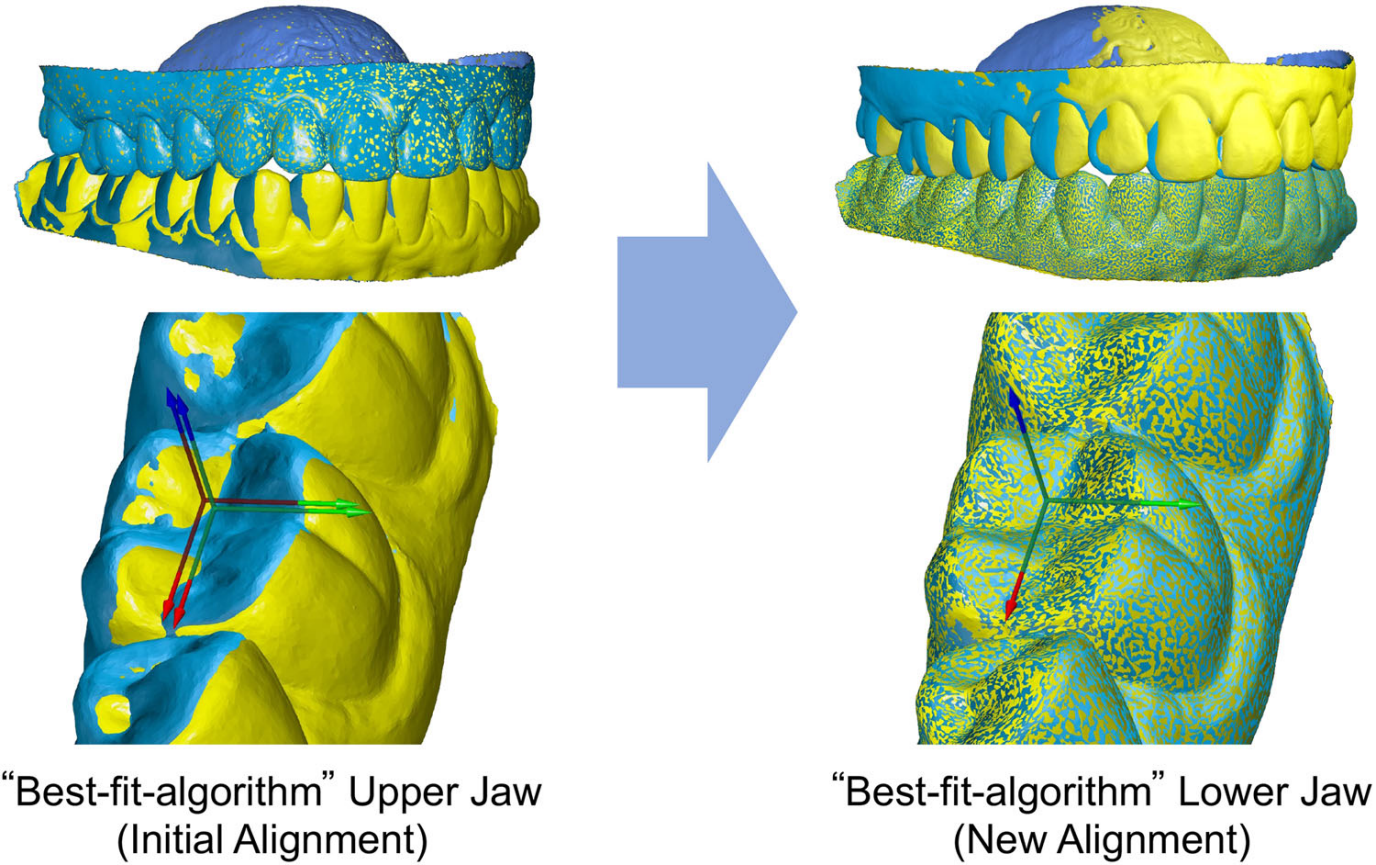
Initial alignment (“Best‐fit‐algorithm” upper jaw) versus new alignment (“Best‐fit‐algorithm” lower jaw).

The first of the two models was defined as the nominal model (scan before therapy/control period; shown in yellow in Figures 2 and 3), while the second model was designated as the corresponding actual model (scan after therapy/control period; shown in blue in Figures 2 and 3).

Subsequently, for the premolars and molars of the lower jaw, a local xyz coordinate system was established based on the nominal model using the 3‐2‐1 method (see Figure 2 on the left side). A corresponding copy of each coordinate system was created for the actual model by the software (see Figure 2 on the right side). In every coordinate system, the *X*‐axis (shown in red in Figure 2) always runs in the mesial‐distal direction, the *Y*‐axis runs in the oral‐vestibular direction (shown in green in Figure 2), and the *Z*‐axis runs parallel to the tooth axis (shown in blue in Figure 2).

Since the exported STL files had different three‐dimensional orientations, an “initial alignment” of the actual model to the nominal model was defined. This “initial alignment” was determined by a local “best‐fit algorithm” in which both upper jaws were congruent (see Figure 3 on the left side). Consequently, the deviations in occlusion result from the three‐dimensional deviation of the lower jaw models in the initial alignment to each other.

To determine these occlusal differences, the two mandibles were now superimposed using a second local “best‐fit algorithm” (see Figure 3 on the right side), and the three‐dimensional displacements of the individual local coordinate systems necessary for this superimposition were recorded by the software. For each local coordinate system (each tooth), the magnitudes of the three‐dimensional displacements in each of the three axes |dX|,|dY|,|dZ| (mm), as well as the Euclidean distance dXYZ (mm), were considered.

### 2.3 Statistical Analysis

Statistical analysis was performed using the statistical programming language R V4.2.2 (R Foundation for Statistical Computing 2022) and RStudio software (RStudio 2022). The Wilcoxon rank sum test was applied as significance test, with a predetermined significance level of 0.05.

To correctly account for the number of subjects of *N* = 10 per group in the statistical analysis, the arithmetic mean of the individual parameters |dX|,|dY|,|dZ|, and dXYZ (mm) was calculated across all teeth of each patient. These are defined as ${\mathrm{dX}}_{\mathrm{Pat}},{\mathrm{dY}}_{\mathrm{Pat}},{\mathrm{dZ}}_{\mathrm{Pat}}$, and ${\mathrm{dXYZ}}_{\mathrm{Pat}}\;\left(\mathrm{mm}\right)$.

## 3 Results

In both groups, the arithmetic mean for the Y‐axis (oral‐vestibular orientation) shows the highest deviations with ${\mathrm{dY}}_{\mathrm{PatMT}}=0.217\pm 0.171\;(\mathrm{mm})$ and ${\mathrm{dY}}_{\mathrm{PatC}}=0.063\pm 0.064\;(\mathrm{mm})$. This is followed by the mean deviations in the *Z*‐axis (parallel to the tooth axis) with ${\mathrm{dZ}}_{\mathrm{PatMT}}\;=\;0.193\pm \;0.131\;(\mathrm{mm})$ and ${\mathrm{dZ}}_{\mathrm{PatC}}\;=\;0.043\pm \;0.024\;(\mathrm{mm})$. On the other hand, in the *X*‐axis (mesial‐distal orientation), the average deviations are lowest with ${\mathrm{dX}}_{\mathrm{PatMT}}=0.122\pm 0.168\;(\mathrm{mm})$ and ${\mathrm{dX}}_{\mathrm{PatC}}=0.060\pm 0.018\;(\mathrm{mm})$.

So, in the arithmetic mean, the subjects of the MT group show higher deviations than the subjects of the control group in all three axes. The difference is significant in the axes *Y* (*p* = 0.0089) and *Z* (*p* = 0.0025) (Table 1).

**Table 1:** TMD short examination according to Ahlers and Jakstat (Caldas et al. 2016).

Examination criteria	Tick as appropriate
Asymmetrical mouth opening	[]
Restricted mouth opening	[]
Joint sounds	[]
Occlusal sounds	[]
Painful muscle palpation	[]
Traumatic eccentricity	[]
TMD	[] Unlikely (≤ 1)
	[] Likely (≥ 2)

The Euclidean distance, which describes the displacement in space in all three dimensions, consequently showed with ${\mathrm{dXYZ}}_{\mathrm{PatMT}}=0.347\pm 0.255\;(\mathrm{mm})$ higher deviations in the subjects treated with manual therapy than in the control group with ${\mathrm{dXYZ}}_{\mathrm{PatC}}=0.113\pm 0.059\;(\mathrm{mm})$. This difference is also significant with *p* = 0.0089.

**Figure 4:**
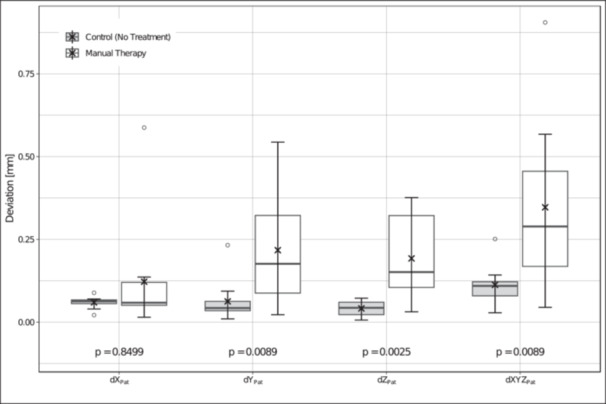
Deviations (mm) in *dX_Pat_*/*dY_Pat_*/*dZ_Pat_*/*dXYZ_Pat_*: Control (No Treatment) versus Manual Therapy (*p*−values were determined using the Wilcoxon rank sum test).

When looking at the boxplot in Figure 4, it is also noticeable that the spread between the minimum (Min) and maximum (Max) within the MT group is always greater than in the control group in all the deviations considered. The same can be seen in Table 2 for the standard deviation (SD).

**Table 2:** Deviations in *dX_Pat_*/*dY_Pat_*/*dZ_Pat_*/*dXYZ_Pat_*: Control (C) versus Manual Therapy (MT) (SD = standard deviation, Min. = Minimum, 1st QU = first quantile, 3rd QU = third quantile, Max. = Maximum, *p*‐values were determined using the Wilcoxon rank sum test).

Deviation axis/group		Mean	SD	*p*‐value	Min.	1st QU	Median	3rd QU	Max.
*dX_Pat_*	C	0.060	0.018	0.8499	0.021	0.056	0.063	0.067	0.089
	MT	0.122	0.168		0.015	0.051	0.059	0.120	0.588
*dY_Pat_*	C	0.063	0.064	0.0089	0.010	0.035	0.043	0.063	0.233
	MT	0.217	0.171		0.023	0.088	0.176	0.322	0.544
*dZ_Pat_*	C	0.042	0.024	0.0025	0.006	0.023	0.043	0.060	0.073
	MT	0.193	0.131		0.031	0.105	0.151	0.322	0.376
*dXYZ_Pat_*	C	0.113	0.059	0.0089	0.028	0.079	0.110	0.122	0.251
	MT	0.347	0.255		0.045	0.169	0.289	0.456	0.905

## 4 Discussion

The aim of the present pilot study was to quantify the changes in occlusion caused by manual therapy compared to a non‐therapy control group.

The results clearly show that manual therapy leads to changes in occlusion in habitual intercuspidation. These changes are significantly greater in the *Y* and *Z* axes and for the Euclidean distance than in the control group that did not receive therapy. The null hypothesis that no changes in occlusion are observed in TMD patients as a result of manual therapy is therefore clearly rejected by the present findings.

This confirms the results of the existing study by Derwich et al. which demonstrated a change in the sagittal as well as vertical position of the mandible in maximum intercuspidation by splint and physiotherapy in TMD patients (Derwich et al. 2022). However, in that study, the observed changes could not be attributed to physiotherapy or splint therapy in a dedicated way, because both therapies were applied immediately one after the other without an intermediate examination (without further FRS).

The changes in the sagittal position of the mandible in that study were recorded using the “wits” parameter, which describes the distance between the perpendicular projection of points A and B onto the functional occlusal plane.

This distance changed by an average of 0.6 mm over the time of the therapies. The sagittal position of the mandible can roughly correspond to the X‐axis (mesial‐distal orientation) of the present study. The deviation of the parameter “wits” falls with 0.6 mm larger than in the present study with ${\mathrm{dX}}_{\mathrm{PatMT}}=0.122\pm 0.168\;(\mathrm{mm})$ and is also statistically not significant.

The vertical position of the mandible was measured by Derwich et al. using the NL‐ML angle, which describes the angle between the nasal line (NL) and mandibular line (ML). Here, significant changes occur with an increase from a mean of 25.5° to 27.1°.

The vertical position of the mandible can be approximately transferred to the changes in the *Z*‐axis (cranial‐caudal) in the present study, which are also significant.

While the observation period of the treated subjects in Derwich et al. is 6 months (Derwich et al. 2022), the intraoral scans in the present study are performed immediately before and after manual therapy with an interval of about 40 min.

In summary (within the limits of comparability of the respective parameters investigated), both studies conclude that sagittal and vertical changes in occlusal relationships can occur as a result of CMD therapies. Derwich et al. attribute the sagittal and vertical changes to the posterior rotation of the mandible, which has also been demonstrated in a previous study (Derwich and Pawlowska 2022).

The arithmetic mean change in occlusion within the control group is ${\bar{\text{dXYZ}}}_{\text{Pat},C1-C2}=0.113\pm 0.059\;\mathrm{mm}$. Therefore, it can be assumed that changes in occlusion may occur even without therapy, since the determined changes are clearly outside usual measurement deviations of the intraoral scanner used (Ender et al. 2016; Ries et al. 2021; Zimmermann et al. 2018). This is also confirmed by an existing study, which reported changes in habitual intercuspidation over the course of the day in healthy patients to be approximately 0.042 mm (Jaschouz and Mehl 2014). Wiechens et al. also conclude in their study that, due to the circadian changes in the topography and intensity of the contact points, occlusion cannot be considered constant (Wiechens et al. 2022).

In contrast, Derwich et al. were unable to demonstrate any sagittal or vertical changes in the position of the mandible in their control group in FRS over a period of one to 2 years (Derwich et al. 2022).

In 2015, Walter et al. performed a pilot study to examine spatial changes in the maxilla following osteopathic treatment in patients with TMD. Impressions of the maxilla were taken both before and after therapy, revealing spatial changes in the maxilla. Moreover, adjustments to the occlusal splint in the lower jaw were necessary posttreatment, indicating a potential alteration in the occlusion (Walter et al. 2015). However, the study's findings are limited due to the small sample size (*N* = 3).

The relationship between occlusion and posture is controversial in the scientific literature. For example, Detoni et al. demonstrated the effects of osteopathic manipulative treatment of the temporomandibular joint on the postural system in patients with TMD (Detoni et al. 2022). Steinmetz et al. also demonstrated that TMD therapy using occlusal splints resulted in a reduction of pain intensity and frequency in professional musicians across various anatomical regions, such as the neck, shoulder and upper extremities (Steinmetz et al. 2009).

Even if the relationship is assumed to be reciprocal, there is more literature data available for an influence of occlusion on posture than vice versa (Manfredini et al. 2012). But in conclusion there is no evidence for an predictable relationship between occlusal and postural features (Manfredini et al. 2012).

However, the present pilot study is limited in its power by some restrictions:

Subjects were randomly selected from the patient population of the student treatment course. The presence of TMD was uniformly determined or excluded on the basis of the TMD brief findings according to Ahlers and Jakstad (Ahlers and Jakstat 2015). However, differentiation according to the different subtypes (e.g., with/without disc displacement, autoimmune components, etc.) was not performed, which is why influences due to this are conceivable.

The average age of all subjects was 31.6 (±15.6) years. The gender distribution was 55% female and 45% male.

The number of subjects with *N* = 10 per group is comparable to other pilot studies also investigating the therapy of TMDs (Alajbeg et al. 2018; Hara et al. 2013). However, verification of the results using larger collectives is desirable (Hara et al. 2013).

Also, the age range from 21 to 77 years is broad and the groups are not sorted by gender, so that influences by gender and age of the subjects cannot be excluded.

Manual therapy was performed in as standardized manner as possible. Thus, all subjects were always treated by the same physiotherapist, who has many years of professional experience as well as special training in the field of temporomandibular joint therapy. Standardized treatment procedures such as mobilization and manipulation of the affected temporomandibular and vertebral joints as well as tender trigger point treatments and stretching of the musculature were applied during the therapy.

After an initial examination of the affected structures in the head and neck area, an individual treatment plan consisting of the above‐mentioned techniques was always compiled and carried out by the therapist at her own discretion.

Digital registration of occlusion using an intraoral scanner has already been used by Jaschouz and Mehl to determine deviations in occlusion (Jaschouz and Mehl 2014). Due to the higher accuracy of habitual intercuspidation registration compared to conventional registration methods (Ries et al. 2021), digital registration of habitual intercuspidation using an intraoral scanner was preferred in the present study.

In addition, digital registration procedures allow significant time savings in comparison to conventional methods, both for virtual impressions of both jaws and for jaw relation determination (Yuzbasioglu et al. 2014).

The scanning was performed with the TRIOS 3 intraoral scanner (3shape, Copenhagen, Denmark).

Zimmermann et al. compared various intraoral scanners with regard to their accuracy in recording habitual intercuspidation (Zimmermann et al. 2018). Here, the TRIOS 3 from the company 3shape showed an translation of 66.6 µm (Zimmermann et al. 2018).

Ries et al. also investigated the accuracy of the registration of occlusion with the TRIOS3 and determined a mandibular deviation of 50 µm on average (Ries et al. 2021).

The occlusion was always recorded while the patient was sitting upright with the head in an upright position to exclude possible influences of the head posture, such as those described for dynamic occlusion (Haralur et al. 2014). However, conversely, it cannot be ruled out that adopting this (as far as possible) standardized posture during the scanning process, has an influence on the occlusion adopted.

To increase the accuracy of the digital registration, care was also taken to include a maximum number of teeth from one half of the jaw when scanning buccally (Revilla‐León et al. 2022).

In summary, this pilot study demonstrated that manual therapy induces significant changes in occlusion in treated TMD patients. The extent to which these changes persist after treatment or how they compare to other TMD therapies (such as splint therapy) remain open questions for future studies.

Although irreversible approaches such as orthodontics and occlusal equilibration are not recommended for TMD management due to a lack of evidence (Durham et al. 2015), it also remains to be determined in following studies whether the observed changes necessitate secondary stabilization using occlusal splints. In certain cases, further orthodontic or prosthetic treatment may be required to achieve long‐term stability and functional harmony within the masticatory system.

## 5 Conclusion

Within the limits of this pilot study, a significant influence on occlusion in habitual intercuspidation can be assumed in TMD patients through manual therapy.

As a clinical consequence, it can be concluded that before initiating prosthetic treatment, TMD therapy using manual therapy should be completed to avoid any influences on the final restoration caused by occlusal changes.

## Author Contributions

Data curation: Tobias Dieter Peter Pohl. Formal analysis: Ragai Edward Matta. Investigation: Tobias Dieter peter Pohl. Methodology: Johannes Ries and Ragai Edward Matta. Project administration: Manfred Wichmann and Ragai Edward Matta. Resources: Manfred Wichmann. Software: Tobias Dieter Peter Pohl. Supervision: Alessia Harhoff, Manfred Wichmann, and Ragai Edward Matta. Validation: Alessia Harhoff. Visualization: Alessia Harhoff and Johannes Ries. Writing – original draft: Tobias Dieter Peter Pohl and Alessia Harhoff. Writing – review and editing: Ragai Edward Matta.

## Ethics Statement

This study was approved by the Clinical Ethics Committee (CEC) at the University Hospital Erlangen (#233_19B).

## Consent

Informed consent was obtained from all individual participants prior their inclusion in the study.

## Conflicts of Interest

The authors declare no conflicts of interest.

## Data Availability

The underlying data is available from the corresponding author upon reasonable request.
